# Behaviour Patterns between Academic Motivation, Burnout and Academic Performance in Primary School Students

**DOI:** 10.3390/ijerph191912663

**Published:** 2022-10-03

**Authors:** Pablo Usán, Carlos Salavera, Alberto Quílez-Robres, Raquel Lozano-Blasco

**Affiliations:** 1 Department of Psychology and Sociology, Faculty of Education, University of Zaragoza, 50009 Zaragoza, Spain; 2 Department of Educational Sciences, Faculty of Humanities and Science Education, University of Zaragoza, 50001 Zaragoza, Spain

**Keywords:** motivation, burnout, academic performance, students, primary education

## Abstract

Background: The final years of primary school (early adolescence) are regarded as key for the academic and personal development of students. This study aims to analyze the relationship between academic motivation, academic burnout, and academic performance, differentiating between adaptive and non-adaptive patterns according to the results of the three constructs studied. Methods: The sample comprised 398 students, both male (*N* = 224; 56.28%) and female (*N* = 174; 43.71%) with ages ranging from 11 to 13 years (M = 11.49; DT = 0.52). The instruments used were the *Maslach Burnout Inventory—Student Survey* (MBI-SS) and the *Academic Motivation Scale* (AMS), as well as academic marks as a measure of performance. Results: The results revealed significant correlations between the three constructs under study, the variables that can be used to predict academic performance, and both adaptive and non-adaptive behavior patterns. Conclusions: The importance of motivation, burnout, and academic performance in primary education is manifested, whose interrelation can give rise to adaptive behaviors based on high school motivation away from academic burnout that leads to a higher academic performance in students.

## 1. Introduction

Currently, in schools, teachers face multiple obstacles to reach their students owing to the large number of personal and academic variables that affect their everyday performance. Teaching requires great degree of commitment, and many teaching professionals feel compelled to adopt a perfectionist and self-demanding attitude in order to respond to the demands posed by their students and society more broadly [1].

In parallel to this, students not only need to adapt to the idiosyncrasies of their teachers, but to a wide array of everyday variables that enhance or undermine their personal and academic skills. These factors, along with their interaction with their peers and family, play a central role in their academic experience [2]. Students perform better when they are motivated and open, engaged in school tasks, and interested in the topics that are being presented in the classroom, leading to adaptive behaviors and a positive personal and academic development [3].

One of the most important variables is academic burnout, defined as the loss of interest in academic topics and the lack of commitment to school tasks, along with self-doubt concerning the student’s ability to meet their academic targets, resulting in stress and poor motivation [4]. The so-called syndrome of academic burnout [5] is characterized by three main dimensions: physical/emotional exhaustion, cynicism (expressed in practices and behaviors that meet widespread disapproval), and personal self-efficacy (self-perception of the ability to meet the academic targets).

The factors that lead to academic burnout are particularly prevalent in the transition from primary to secondary education, resulting in low levels of academic wellbeing and performance, potentially leading to school dropout [6]. Previous studies have related academic burnout with high levels of stress [7]; low levels of academic self-efficacy [8]; low academic motivation and performance [9]; and low levels of personal and academic happiness [10].

Another important variable for learning is motivation. Today, motivation is a recurrent factor in explanations concerning varying levels of school performance. In order to learn something new, it is necessary to have the required skills, knowledge, and strategies, but also the predisposition and will to learn [11]. It is for this reason that motivation plays such an important role in any tasks involving the acquisition, transference, and use of knowledge [12]. Academic motivation has been paid a good deal of scholarly attention. Self-determination theory (SDT) [13] is a macro-theory of human motivation which assumes persons as active organisms that integrate their experiences consistently. SDT defines a continuum that spans several degrees of self-determination, which are internally or externally regulated by the subject and defined in terms of intrinsic motivation, extrinsic motivation and amotivation. Intrinsic motivation refers to initiating an activity because it is satisfying to do so, without the need for any extrinsic stimuli, and can be directed towards knowledge, achievement, and pleasant experiences. Extrinsic motivation is geared towards a goal and can be divided into externally regulated behavior, introjected regulation, and regulation through identification. Amotivation refers to the lack of motivation in a given task.

In this way, academic motivation is a key factor in terms of academic commitment, as it represents the will and conscious decision of students to actively engage in their tasks [14]. Previous studies have related academic motivation with academic effort [15]; academic self-efficacy [16]; emotional intelligence [17]; school dropout [18]; and, more generally, academic wellbeing [19].

Finally, academic performance is the level of academic achievement, understood both quantitatively and qualitatively [20]. It is not uncommon to mistake effort for skill, e.g., when the level of effort invested does not reflect on results. For this reason, we must focus on the relationship between effort and skill, identifying situations in which skill will play a greater role than effort (when results come more ‘easily’) and those in which skills lag behind effort, leading to negative results, overwhelming the student, and causing anxiety owing to the unbalance between effort invested and outcome [1].

Therefore, academic performance is a multidimensional construct constituted by multiple variables. Fierro et al. [21] argue that motivation and emotional intelligence can be used to predict academic performance [22] focus on individual personality traits; and Pulido and Herrera [23] argue for the effect of socio-demographic variables. In any case, school marks are the most efficient and more stable way to measure academic performance [24,25] although other methods have been suggested, such as the number of failed subjects, the number of repeats, and even the time invested in learning [26,27].

There are hardly any studies that directly relate the constructs of our study and there are fewer studies that relate them in primary education; hence one of the novelties of this study. Trigueros et al. [28] in a sample of university students, related demanding academic situations and the use of inadequate coping strategies as the main reasons for dropping out of school. In addition, it was deduced that academic motivation negatively predicted burnout and positively predicted academic performance, while burnout negatively predicted school performance. Dogan [29], in a sample of adolescent high school students, found that academic self-efficacy and school motivation predicted students’ academic performance. Additionally, students’ self-ability motivation and related motivations, as well as their sense of purpose in learning, are significant variables that will result in their academic success. Finally, Asakereh and Yousofi [30] in a sample of students in upper secondary education, re-consolidate the relationship between academic motivation and academic performance as well as the negative relationship between this and burnout. At the same time, the regression results demonstrated that academic attrition had a better ability to predict academic performance compared to intrinsic motivation in students.

In this context, the main aim of this study is to examine the relationship between academic motivation, burnout, and academic performance in students in the two final years of primary education. We define adaptive behavior as the set of conceptual, social, and practical skills that the individual has learned and that allow him to respond to the circumstances of daily life. By non-adaptive behavior, we understand socially annoying or disturbing behaviors that prevent or reduce the possibilities of adaptation to the environment, as well as the development or new academic and social behaviors.

The four starting hypotheses are as follows:

**Hypothesis** **1.** Academic motivation, burnout, and academic performance are closely related.

**Hypothesis** **2.** Adaptive behavior patterns are characterized by high intrinsic motivation and academic performance and low academic burnout and amotivation.

**Hypothesis** **3.** Non-adaptive behavior patterns are characterized by high extrinsic motivation, burnout, amotivation, and poor academic performance.

**Hypothesis** **4.** Intrinsic and extrinsic motivation, amotivation and burnout can be used to predict academic performance.

## 2. Method

***Sample:*** The sample comprised 398 students, both male (*N* = 224; 56.28%) and female (*N* = 174; 43.71%) in the two final years of primary education in Zaragoza, Aragón, Spain. The schools selected to carry out the study were chosen by random sample. The data were collected in January and February 2022. All questionnaires were anonymous, and all participants were volunteers.

***Instruments:*** Burnout was measured using the Spanish version of the *Maslach Burnout Inventory—Student Survey* (MBI-SS) [5]. This is a 15-item questionnaire which comprises three dimensions: physical/emotional exhaustion, cynicism, and self-efficacy. Answers are provided in a 5-point Likert scale ranging from ‘Totally disagree’ (1) to ‘Totally Agree’ (5). The original questionnaire yields a Cronbach’s alpha value of 0.81 and 0.83 in our study.

Academic motivation was measured using the *Academic Motivation Scale* (AMS), adapted for the academic context [11]. This is a 28-item questionnaire which comprises three dimensions: intrinsic motivation (knowledge, achievement, and stimulating experiences); extrinsic motivation (external, identified, and introjected), and amotivation. Answers are provided in a 5-point Likert scale ranging from ‘Totally disagree’ (1) to ‘Totally agree’ (5). The original questionnaire yields a Cronbach alpha value of 0.80, and 0.81 in our study.

Academic performance was measured as the average marks of students after the first school term, ranging from 0 to 10. This is one of the most widely used and reliable methods available to measure academic performance [24,25]. This variable yielded a Cronbach alpha value of 0.86 in our study.

***Procedure:*** The questionnaires were handed out in the classroom with the authorization of the school management and the parents/tutors, who signed an informed consent form. The study followed all the ethical guidelines set out in the Declaration of Helsinki [31]. The study protocol was endorsed by group OPIICS (S46_20R), Psychology and Sociology Department, University of Zaragoza.

***Data analysis:*** Statistics were collected to establish the socio-demographic variables of the sample, including gender, age, kind of school, and the averages of these variables. Afterwards, SPSS v25 software was used to carry out Pearson correlation analyses on the scales, since they are numerical, scalar, and continuous, in order to establish the relationship between academic motivation, burnout, and academic performance (Hypothesis 1). Secondly, cluster analysis K-medias was used to divide the sample into three significant groups. Clustering is the process of dividing all the data into three large groups, as the number most used in the scientific literature that allows us to verify the behavioral profiles under investigation, also known as clusters, based on the patterns of all the data of the investigation (Hypothesis 2 and 3). Finally, this was followed by regression analysis (‘introduction’ method) using academic performance as the criterion variable over the other two dimensions (Hypothesis 4). For all the operations, a *p* ≤ 0.05 level of significance was adopted, with a 95% confidence level.

## 3. Results

### 3.1. Sociodemographic and Descriptive Variables

The sample comprised 398 primary school students, both male (*N* = 224; 56.28%) and female (*N* = 174; 43.71%) with ages ranging from 11 to 13 years (M = 11.49; DT = 0.52) (Table 1).

Concerning the descriptive variables of the study (Table 2), self-efficacy (the most adaptive dimension of burnout) yielded a (small) significant size effect, especially in girls (Cohen’s *d* = −0.220), whereas amotivation was found to be higher among boys (Cohen’s *d* = −0.280).

### 3.2. Correlation Analysis

Correlation analyses (Table 3) demonstrated that the three constructs under analysis are closely related. Amotivation was shown to be positively correlated with the two less self-determined dimensions of burnout: exhaustion (*r* = 0.535) and cynicism (*r* = 0.395), as well as with some extrinsic motivations, and negatively correlated with predominantly intrinsic motivations. Intrinsic motivations were also shown to correlate positively with one another, and with the most self-determined dimension of burnout: self-efficacy. Finally, academic performance was shown to be positively correlated with intrinsic knowledge motivation (*r* = 0.160) and achievement (*r* = 0.277), and with self-efficacy (*r* = 0.310).

### 3.3. Cluster Analysis in Significant Groups

K-means cluster analysis was undertaken to divide the sample into three significant groups: group 1 (*N* = 42; 10.55%); group 2 (*N* = 128; 32.16%); and group 3 (*N* = 228; 57.28%). As presented in Table 4, group 2 is characterized by near-average values in all variables. Group 1 is characterized by non-adaptive behaviors, reflected by above average values in terms of burnout and amotivation, and below average values in terms of intrinsic motivation and academic performance. Group 3 is characterized by adaptive behavior, dominated by above average values in terms of intrinsic and extrinsic motivations and below average values in terms of academic burnout.

### 3.4. Regression Analysis

Finally, regression analysis by the introduction method was undertaken, selecting the factorial scores for academic motivation and academic burnout as predicting variables and academic performance as the criterion variable. Table 5 presents the significant predictive variables. The most valuable variables in terms of prediction were the most self-determined variable of burnout (self-efficacy), intrinsic achievement motivation, and amotivation. Nagelkerke’s *R*^2^ statistics yielded an adjustment value of 0.289 to explain the model.

## 4. Discussion

The main aim of the study was to investigate the relationship between the three constructs under consideration: academic motivation, burnout, and academic performance. In this section, we shall compare our results with our hypotheses and the results of previous studies.

The first hypothesis held that academic motivation, academic burnout, and academic performance are closely related. This hypothesis was fully confirmed, as shown by the bi-directional correlations between specific dimensions. These results link with our remaining hypotheses.

The second hypothesis held that adaptive behaviors are characterized by high intrinsic motivation and academic performance and low academic burnout and amotivation. Our results largely support this hypothesis.

Previous studies point out the relationship between intrinsic motivation and academic performance. Ferriz et al. [32] emphasize the relationship between intrinsic motivation and good school marks. Cuevas et al. [33] relate intrinsic motivation to satisfaction and happiness, concluding that intrinsic motivations have a direct impact on academic performance in secondary school. Froiland & Worrell [34] connect intrinsic achievement motivation, intrinsic motivations, and academic engagement with academic performance in secondary school students. Suárez & Suárez [35] argue that intrinsic motivation plays a key role in the assumption of effective learning skills, leading to higher levels of academic self-efficacy.

Concerning the relationship between intrinsic motivation and burnout, the literature is less clear. While our results suggests an unequivocal correlation (the more motivated a student is, the least physical/emotional exhaustion and cynicism they feel), a result supported by most previous studies [36,37,38], other studies are less certain, finding that some intrinsically motivated students suffer exhaustion owing to one of the dimensions of academic burnout [39,40].

Finally, intrinsic motivation and amotivation, the two opposite ends of the SDT spectrum, were found to be negatively correlated [13], as also pointed out by previous studies [41,42,43].

Our third hypothesis held that non-adaptive behavior patterns are characterized by high extrinsic motivation, burnout, amotivation, and poor academic performance. Our results partially support this hypothesis.

First, no significant relationship was found between extrinsic motivation and academic performance. Some previous studies argue for a direct relationship between extrinsic motivations and poor school results. Baena et al. [44], for instance, concluded that these constructs were positively correlated. Liu et al. [45] reached similar conclusions after a lateral study with a student sample. Moreover, Salanova et al. [46] also take into consideration the burnout dimensions of emotional exhaustion and cynicism, finding a positive correlation with poor school performance. In contrast, other works do not find a relationship between academic performance and predominantly extrinsic motivation. Tsouloupas et al. [47] argue that extrinsic motivation has no significant effect on academic performance; and McCollum and Kajs [48] find no link between extrinsic motivation and goal orientation and academic performance. This may be due to the fact that some students are intrinsically and extrinsically motivated simultaneously, using one or the other orthogonally depending on the task at hand and/or on personal variables [49].

Second, our results do not suggest a direct relationship between extrinsic motivation and academic burnout. Barbosa et al. [50] also argue against this relationship, and even argue for a motivational profile in which extrinsic motivation does not correlate with academic burnout or academic performance. We must, however, be cautious, because, as pointed out by Jaramillo [51] students can skillfully and non-contradictorily handle both kinds of motivation (owing to their orthogonal nature) to cope with everyday challenges [52].

Third, the relationship between extrinsic motivation and amotivation was not confirmed. This means that extrinsic motivation does not necessarily crystallize in lack of academic motivation and poor school results. Tsouloupas et al. [47] argue that extrinsic motivation is related to academic performance, but that this relationship has no significant impact on school results. McCollum and Kajs [48] find a link between both extrinsic and intrinsic motivation and school performance. Finally, Delgado et al. [53] allude to variables other than extrinsic motivation, such as gender and age, to explain academic performance. Following these studies, it can be concluded that extrinsic and intrinsic motivations do not have a dichotomic and contradictory relationship but are dimensions of the same pattern of behavior. Some students can be motivated both intrinsically and extrinsically, using one or the other orthogonally, depending on the task at hand and/or personal variables [49]. Extrinsic motivation, therefore, must not always be negatively assessed [54].

Finally, our results point to a negative correlation between academic burnout and academic performance. This hypothesis is divided into two sections. First, taking the subvariables of our study, previous studies emphasize this relationship, and some even claim that burnout is also negatively correlated with emotional intelligence. Caballero et al. [4] and Salanova et al. [46] reach a similar conclusion, arguing that students that present high levels of physical and emotional exhaustion and cynicism tend to perform poorly. Evers et al. [55] present a similar argument, and emphasize the enhancing effect of social networks. Madigan and Curran [56] also found this relationship in a study with a large student sample. Finally, Naderi et al. [57] stress that high burnout and poor academic performance are often accompanied by low self-efficacy.

Secondly, referring to Bresó et al. [58] other studies taking the general constructs of burnout and academic performance follow the same line in terms of the general prevalence of both constructs maintaining a negative relationship. Usán et al. [9], in a sample of adolescent students, mention high levels of emotional intelligence together with high academic performance compared to students with low levels of academic burnout. Comella et al. [59], in a sample of university students who combined presence at the University with their work tasks, find significant relationships between both general constructs regardless of whether they work or not, appreciating an increase in burnout as advancing at the University. Finally, Chavergen [60] determines the inversely proportional relationship between burnout syndrome and academic performance in a sample of final year university students.

Finally, our fourth hypothesis held that intrinsic and extrinsic motivation and burnout can be used to predict academic performance. The hypothesis was partially confirmed as the number of predictive variables found was lower than expected. Few previous studies have directly addressed this issue. Kumar and Tankha [61] establish achievement motivation, along with educational adjustment, as a predictive variable of academic performance, leaving aside aspects related to emotional intelligence. Steinmayr et al. [62] also relate achievement motivation, alongside other forms of intrinsic motivation, to school results. Ozkal [63] emphasizes the important role played by self-efficacy and purely intrinsic motivations in school performance, in line with our results. Asakereh and Yousofi [30] stress the importance of self-efficacy, self-esteem, and reflexive thought for academic performance. Finally, Ramos et al. [64] point out the relationship between self-efficacy and motivation and school performance.

## 5. Conclusions

Based on our result, the importance of variables, such as motivation, academic burnout, and academic performance, for the adoption of adaptive and non-adaptive behaviors in primary school students cannot be overemphasized.

The limitations of the study include the fact that the data were collected at a given point in time, so the evolution of variables depending on the personal circumstances of students cannot be tracked over time. Moreover, the schools were chosen by random sampling, instead of reflecting a stratified and representative sample, so results may be affected by sampling bias. In practical terms, the study should encourage teachers to implement practices to prevent academic burnout and improve performance and, with this, support a more satisfactory academic and personal life for their students. Similarly, educational programs should cultivate the psychological variables that lead to the harmonious development of the students’ skills and their personal and academic development.

For the future, it would be interesting to pay specific attention to other factors that have a direct impact on the academic performance of students, and even examine variables which contribute to increase academic motivation and prevent burnout in more depth. Furthermore, longitudinal studies could help to understand the evolution of these psychological variables over time, including other educational stages.

## Figures and Tables

**Table 1:** Socio-demographic variables.

		*N*	%
**Gender**	Boys	224	56.28
	Girls	174	43.71
**Age**	11 years	198	49.74
	12 years	186	46.73
	13 years	14	3.51
**Academic year**	5º PRI	192	48.24
	6º PRI	206	51.75
**Type of school**	Public	274	68.84
	Private	124	31.15

**Table 2:** Descriptive variables.

	*Total*		*Male*		*Female*		
	*x*	*sd*	*x*	*sd*	*x*	*sd*	*Cohen’s d*
**Burnout**							
1. Exhaustion	2.40	0.69	2.45	0.74	2.30	0.60	0.110
2. Cynicism	1.99	0.67	2.02	0.68	1.93	0.66	0.069
3. Self-efficacy	3.93	0.60	3.84	0.64	4.09	0.45	−0.220
**Motivation**							
4. Intrinsic knowledge	3.84	0.87	3.78	0.97	3.93	0.63	−0.091
5. Intrinsic achievement	3.47	0.86	3.36	0.90	3.67	0.76	−0.182
6. Intrinsic stimulating experiences	3.07	0.92	3.12	0.95	2.96	0.88	0.087
7. Extrinsic external regulation	4.01	0.75	4.06	0.74	3.90	0.76	0.106
8. Extrinsic introjected regulation	3.58	1.01	3.56	1.03	3.60	0.98	−0.019
9. Extrinsic identified	4.31	0.60	4.30	0.62	4.32	0.55	−0.017
10. Amotivation	1.89	0.88	2.06	0.99	1.60	0.51	0.280
**Academic performance**							
11. Average mark	3.22	1.25	3.17	1.31	3.30	1.15	−0.052

**Table 3:** Correlation analysis.

	1	2	3	4	5	6	7	8	9	10	11
**Burnout**											
1. Exhaustion	1										
2. Cynicism	0.483 **	1									
3. Self-efficacy	−0.557 **	−0.437 **	1								
**Motivation**											
4. Intrinsic knowledge	−0.359 **	−0.161	0.524 **	1							
5. Intrinsic achievement	−0.258 *	−0.120	0.504 **	0.732 **	1						
6. Intrinsic stimulating experiences	−0.161	−0.104	0.322 **	0.641 **	0.507 **	1					
7. Extrinsic external regulation	−0.67	0.023	0.082	0.133	0.239 *	0.184	1				
8. Extrinsic introjected regulation	−0.148	0.023	0.289 **	0.363 **	0.578 **	0.328 **	0.573 **	1			
9. Extrinsic identified	−0.298 **	−0.085	0.255 *	0.417 **	0.309 **	.275 **	0.577 **	0.281 **	1		
10. Amotivation	0.535 **	0.395 **	−0.555 **	−0.606 **	−0.514 **	−0.184	−0.164	0.258	0.404 **	1	
**Academic performance**											
11. Average mark	−0.047	−0.099	0.310 **	0.160 *	0.277 **	0.196	0.097	0.103	0.117	0.160	1

** The correlation is significant at 0.01 (bilateral). * The correlation is significant at 0.05 (bilateral).

**Table 4:** Cluster analysis.

	*Group 1*	*Group 2*	*Group 3*	*Total*		
	*x*	*x*	*x*	*x*	*F*	*Sig.*
**Burnout**						
1. Exhaustion	3.33	2.31	2.26	2.40	11.574	0.000
2. Cynicism	2.42	2.02	1.82	1.99	2.812	0.067
3. Self-efficacy	3.00	3.85	4.23	3.93	23.165	0.000
**Motivation**						
4. Intrinsic knowledge	2.08	3.79	4.23	3.84	45.321	0.000
5. Intrinsic achievement	2.19	3.25	4.06	3.47	33.666	0.000
6. Intrinsic stimulating experiences	2.00	2.88	3.51	3.07	13.986	0.000
7. Extrinsic external regulation	3.89	3.95	4.18	4.01	1.026	0.364
8. Extrinsic introjected regulation	2.56	3.36	4.10	3.58	13.467	0.000
9. Extrinsic identified	3.83	4.31	4.54	4.31	6.225	0.003
10. Amotivation	3.61	1.68	1.56	1.89	45.810	0.000
**Academic performance**						
11. Average mark	2.89	2.10	4.17	3.22	45.647	0.000

**Table 5:** Regression analysis.

	*B*	s.e.	*R* ^2^	*t*	Sig.
**Constant**	−3.794	2.313	0.342	−1.640	0.106
Exhaustion	0.226	0.274		0.825	0.413
Cynicism	−0.314	0.243		−1.292	0.201
Self-efficacy	0.756	0.333		2.270	0.027
Intrinsic knowledge	−0.172	0.344		−0.499	0.620
Intrinsic achievement	0.626	0.259		2.421	0.018
Intrinsic stimulating experiences	0.131	0.216		0.606	0.546
Extrinsic external regulation	−0.135	0.285		−0.474	0.637
Extrinsic introjected regulation	0.056	0.210		0.268	0.790
Extrinsic identified	0.295	0.339		0.869	0.388
Amotivation	0.643	0.271		2.373	0.021

## Data Availability

Data available asking authors.
